# Supplementary material for: Nasopharyngeal Expression of Angiotensin-Converting Enzyme 2 and Transmembrane Serine Protease 2 in Children within SARS-CoV-2-Infected Family Clusters
Source: Microbiol Spectr. 2021 Nov 3;9(3):e00783-21. doi: 10.1128/Spectrum.00783-21 (PMC8567246; doi:10.1128/Spectrum.00783-21)
Supplement: SUPPLEMENTAL FILE 1 — Supplemental material. Download SPECTRUM00783-21_Supp_1_seq6.pdf, PDF file, 0.1 MB [file spectrum00783-21_supp_1_seq6.pdf]

Supplemental Information

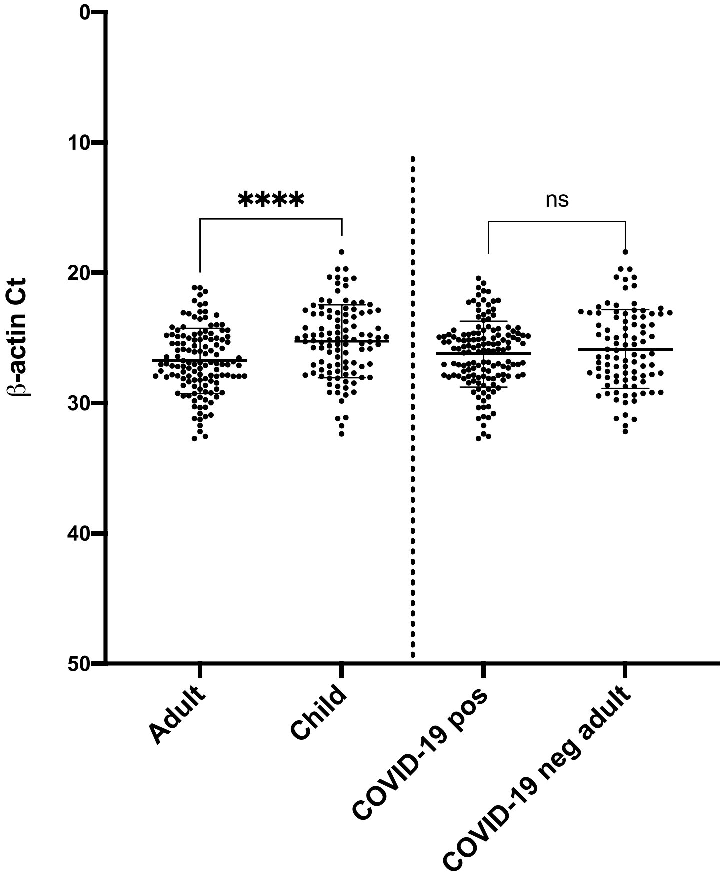

Supplementary Figure 1: Comparison of β-actin gene expression between adults and children and between SARS-CoV-2 positive and negative participants. Mean with 95% CI of qPCR Ct values for β-actin gene in different groups. *p*-values were calculated by unpaired 2-tailed T test; **** = $p<0.0001$, ns = not significant.

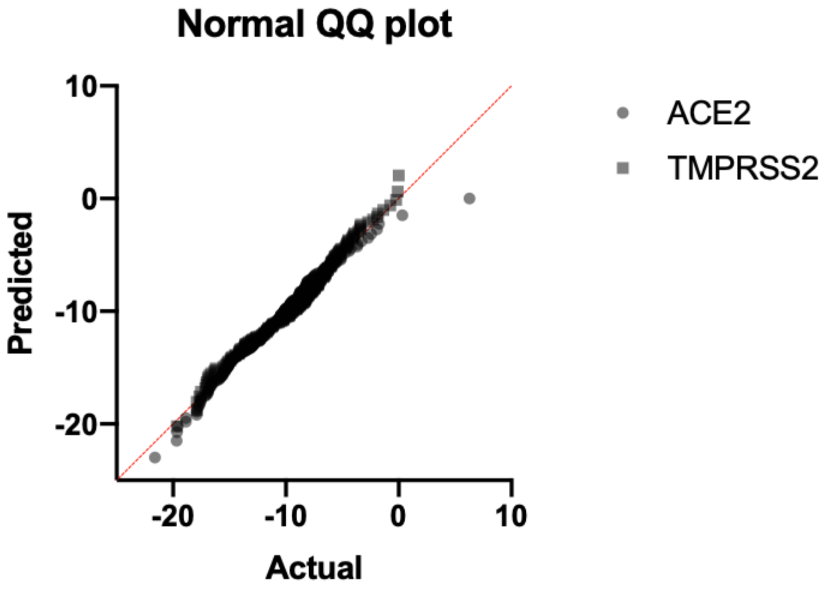

Supplementary Figure 2: Normality test for ACE2 and TMPRSS2 transcript levels. The distribution of qPCR Ct values relative to β-actin for ACE2 and TMPRSS2 was tested by Shapiro-Wilk test (W=0.9822; p=0.0049 for ACE2 and W=0.9807; p=0.0028 for TMPRSS2) and Anderson-Darling test (A2=0.58; p=0.1301 for ACE2 and A2=1.774, p=0.0001 for TMPRSS2).

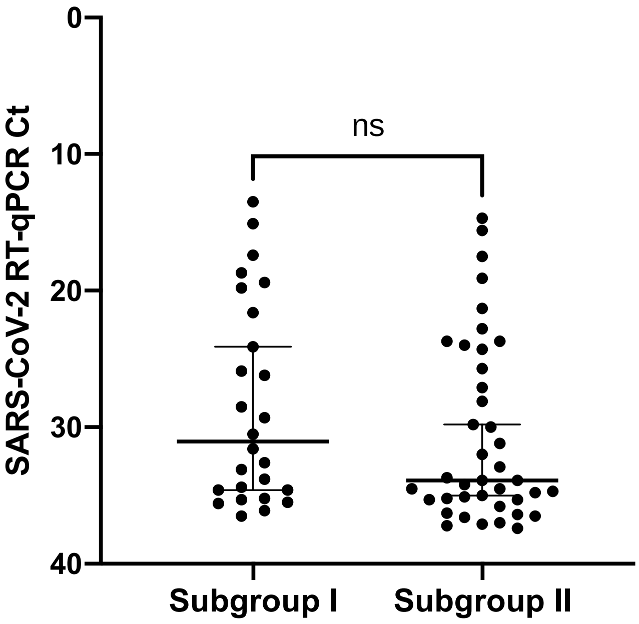

Supplementary Figure 3: Comparison of viral load in SARS-CoV-2 positive adults from families with SARS-CoV-2 negative (Subgroup I) versus positive (Subgroup II) children. Median with 95% CI of RT-qPCR Ct values for SARS-CoV-2 in different groups. *p*-values were calculated by Mann-Whitney U test; ns = not significant. Mean Ct values between the two groups were also compared by unpaired 2-tailed T test and were not significantly different from each other (p=0.2166). ns, not significant.

Supplementary Table 1: Nasopharyngeal ACE2 and TMPRSS2 gene expression in COVID-19 positive versus negative patients

Population	Gene	#Median (IQR)		*p-value
		COVID-19 positive	COVID-19 negative	
Overall	ACE2	-11.02 (-14.24 to -8.413)	-12.31 (115.38 to -9.702)	0.0110
	TMPRSS2	-8.191 (-11.19 to -6.063)	-9.051 (-12.97 to -6.506)	0.1364
Adult	ACE2	-10.91 (-13.0 to -8.176)	-11.35 (-13.75 to -9.378)	0.1603
	TMPRSS2	-8.242 (-10.40 to -5.997)	-8.375 (-10.7 to -6.611)	0.6810
Children	ACE2	-11.45 (-15.2 to -8.82)	-12.80 (-16.42 to -10.44)	0.0601
	TMPRSS2	-8.029 (-11.67 to -6.237)	-10.15 (-13.74 to -6.414)	0.1630

*p-values were calculated by two-tailed, Mann-Whitney U test

#ΔCt values were calculated by subtracting the Ct values for ACE2 or TMPRSS2 from the respective Ct values for the housekeeping gene β-actin.

Supplementary Table 2: Association of detectable, nasopharyngeal ACE2 and TMPRSS2 gene expression (RT-qPCR Ct < 40) with SARS-CoV-2 infection

Group	Gene	Sample no.	Adjusted for	OR (95% CI)	*p*-value
Overall	ACE2	99	Age and gender	1.146 (0.994-1.337)	0.0651
		99	Gender	1.159 (1.014-1.351)	**0.0419**
		99	None	1.158 (1.013-1.350)	**0.0434**
	TMPRSS2	191	Age and gender	1.022 (0.9352-1.118)	0.6293
		191	Gender	1.030 (0.9436-1.126)	0.5059
		191	None	1.030 (0.9437-1.126)	0.5043
Families with at least one adult positive for COVID-19	ACE2	77	Gender	1.180 (1.041-1.366)	**0.0159**
		77	None	1.198 (1.059-1.380)	**0.0072**
	TMPRSS2	142	Gender	1.113 (1.001-1.245)	0.0526
		142	None	1.150 (1.043-1.277)	0.0066
Families with adults negative for COVID-19	ACE2	25	Gender	0.8341 (0.5525-1.169)	0.3252
		25	None	0.8253 (0.5823-1.100)	0.2165
	TMPRSS2	50	Gender	0.8047 (0.6543-0.9599)	**0.0234**
		50	None	0.8487 (0.6996-1.001)	0.0673

OR, odds ratio; CI, confidence intervals. OR calculated from multiple logistic regression analysis
